# *Aspergillus *Section* Nigri*-Associated Calcium Oxalate Crystals in an Eurasian Eagle Owl (*Bubo bubo*)

**DOI:** 10.1155/2018/3807059

**Published:** 2018-06-12

**Authors:** Patti K. Kiser, Danielle M. Meritet, Robert J. Bildfell

**Affiliations:** ^1^Department of Biomedical Sciences, College of Veterinary Medicine, Oregon State University, Corvallis, OR, USA; ^2^Oregon Veterinary Diagnostic Laboratory, Oregon State University, Corvallis, OR, USA

## Abstract

An adult male Eurasian eagle owl (*Bubo bubo*) housed at a wildlife rehabilitation facility in southern Oregon died after a short period of progressive ill-thrift. Radiographs taken prior to death demonstrated abnormal radiopaque material in the coelom and the owl was submitted for postmortem examination. Black pigmented fungus was noted grossly, particularly in the respiratory tissues, with abundant oxalate crystal deposition associated with and without hyphal elements subsequently observed histologically.* Aspergillus *section* Nigri *was cultured from the lesions. Although there have been a few reports of aspergillosis caused by* Aspergillus niger* in avian species, the severity and wide tissue distribution of oxalates in this case are highly unusual.

## 1. Introduction

Aspergillosis is a relatively common respiratory pathogen of avian species. Oxalate crystal formation by* Aspergillus* species has been documented in both avian and nonavian hosts, often at the site of fungal colonization (i.e., lungs, air sacs) or at sites of their excretion (i.e., kidney). This case report describes a unique distribution of oxalate crystal formation due to an* Aspergillus *section* Nigri* infection in a Eurasian eagle owl.

## 2. Case Presentation

**Figure 1:**
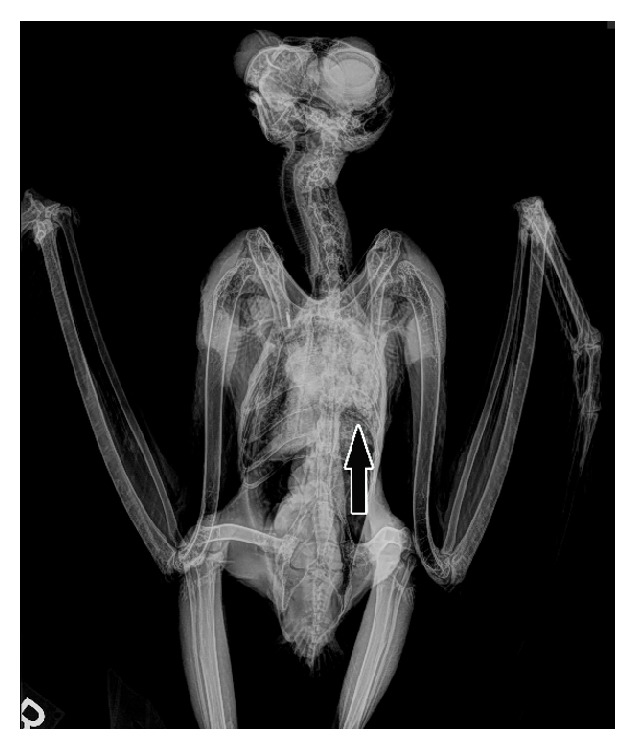
Eurasian eagle owl, ventrodorsal radiographs. Increased soft tissue opacity in left lung (arrow).

An adult male Eurasian eagle owl housed at a wildlife rehabilitation facility in Southern Oregon State was presented to a veterinary clinic with a history of one week of progressively worsening dyspnea, inappetence, and lethargy. Approximately 10 days earlier, the bird had sustained minor lacerations due to an attack by its female mate, and several months before the bird had undergone ocular surgery to correct trauma-associated lenticular rupture of the left eye. Workup of the dyspnea problem included radiographs which revealed increased areas of soft tissue opacity within the coelom, especially in the lung fields (Figure 1). Potential differentials for increased soft tissue opacity within the lung included pneumonia, fluid (e.g., edema, hemorrhage), and neoplasia. Bloodwork was also performed revealing a heterophilia (13.12x10^9^/L, range 5.8-9.8 x10^9^/L), hyperglobulinemia (39g/L, range 0-35g/L), hyperphosphatemia (5.46mmol/L, range 0.19-3.1mmol/L), hyperkalemia (7.40mmol/L, range 0.90-5.00mmol/L), and a mild elevation in creatine phosphokinase (20.37*μ*kat/L, range 0-18.04*μ*kat/L). The bird was returned to its enclosure following these diagnostic tests but found dead the next day. The remains were submitted for postmortem examination at the Oregon Veterinary Diagnostic Laboratory.

**Figure 2:**
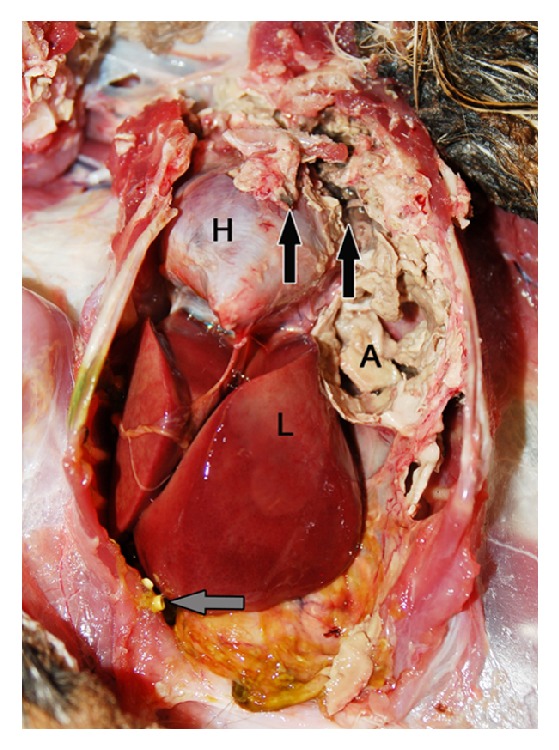
Eurasian eagle owl, coelomic cavity. The abdominal air sac (A) thickened by friable tan to grey exudate. The caudal aspect of the thoracic air sacs coated by black fuzzy material (black arrows). Numerous 1-2 mm yellow-tan plaques on the serosal surfaces of the abdomen (grey arrow). The heart (H) and liver (L) are labeled for anatomic orientation.

The 1.5 kg bird was in poor body condition (body condition score of 1/5) but had excellent quality of plumage. The left eye had pupillary distortion and slight corneal clouding from a healing incision. On gross examination of the coelom, the left thoracic air sacs were thickened by friable grey exudate and collapsed upon manipulation, revealing lumina filled with brown friable debris that extended into the subjacent pulmonary parenchyma (Figure 2). This necrotizing and granulomatous pulmonary change was present bilaterally but was more severe on the left side with approximately 50% of this lung field compromised. The caudal aspect of the thoracic air sacs was coated by a layer of black fuzzy material, confirmed on impression smear to be fungal spores. Yellow, firm plaques of exudate covered the outer surface of the tracheal bifurcation and the cranial serosal aspect of the proventriculus. Dozens of 1-2 mm raised yellow-tan, less sharply defined foci, sometimes with a fuzzy surface, covered the serosal surfaces of abdominal (Figure 2) and cranial thoracic viscera and the lining of cervical air sacs.

**Figure 3:**
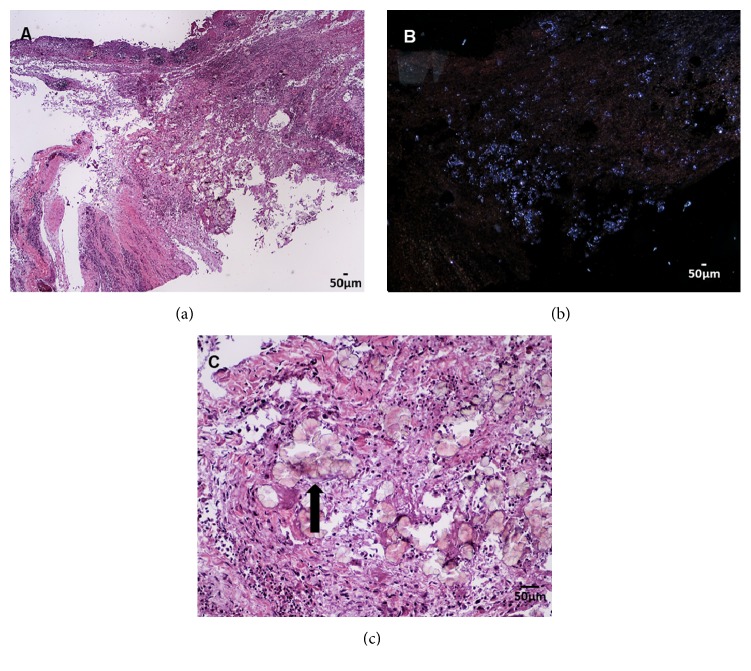
Eurasian eagle owl, air sac. (a),(b) The air sac wall was diffusely thickened by an exudate of fibrin, heterophils, and fewer macrophages with entrapped oxalate crystals. H&E stained section (a) with comparable view under polarized light (b). Polarized light with lower light intensity to better visualize the birefringent, entrapped oxalate crystals. Original objective 4x. (c) Oxalate crystals entrapped within the air sac wall (arrow). Crystals have the characteristic wheat sheaf and rosette arrangement for calcium oxalate. Original objective 20x.

**Figure 4:**
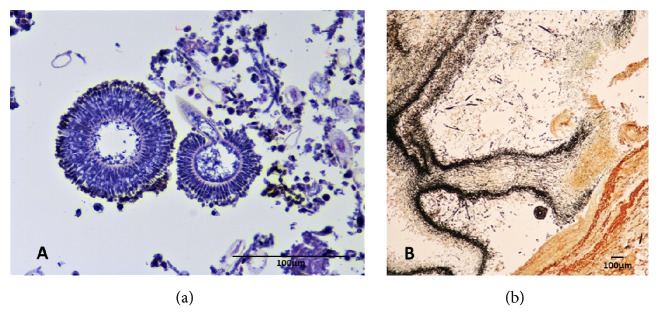
Eurasian eagle owl, air sac. Pigmented conidial heads mixed with fungal hyphae. (a) Visualization of conidiophores of* Aspergillus* section* Nigri* on routine H&E section. Original objective 40x. (b) Fungal mats characterized by dark black outlines with Grocott's methenamine silver (GMS) stain. Original objective 40x.

Using a Leica DM 1000 light microscope, examination of hematoxylin and eosin stained histology slides revealed the abdominal air sac wall was diffusely thickened by an exudate of fibrin, heterophils, and fewer macrophages with entrapped oxalate crystals (Figure 3). The oxalate crystals were characterized by translucent, pale yellow, highly birefringent, anisotropic linear shapes arranged in sheaves and rosettes (Figure 3). The surface of the air sac was colonized by dichotomously branching fungal hyphae with parallel walls. Fruiting bodies (conidiophores) were visible, including the stalk (variably present), central vesicle with circumferentially biseriate, radiating linear metulae and phialides, and peripheral round, pigmented conidia (Figures 4(a) and 4(b)) and Grocott's methenamine silver stain rarely demonstrated septae.

Similar to the air sac, fungal bodies and oxalate crystals were present within other respiratory tissues. The examined lung tissue contained large areas of coagulation necrosis with a portion composed almost entirely of fungi, including fruiting bodies. Hemorrhage into airways was common with some occupied by fibrin. Abundant crystals were present, especially in the walls of arteries, and thrombosis was common. Granulomatous inflammation was occasionally noted. The tracheal mucosa was multifocally eroded with heterophils and mucus coating the surface. The exposed tracheal stroma contained many rosettes of birefringent light yellow crystals, as well as some macrophages, heterophils, and plasma cells.

Remarkably, oxalate crystals were present in other organs, lacking fungal elements. The small intestine crypts were dilated and filled with protein rich fluid, erythrocytes, and heterophils mixed with occasional oxalate crystals. In some areas of the small intestine with a clearly intact mucosal epithelium, oxalate crystals were deposited in the lamina propria of a villus tips and in the wall of submucosal arterioles. The ventriculus contained a few rosettes of crystals within the koilin layer. Cardiac, cerebellar, and hepatic small caliber blood vessels were expanded by birefringent crystals.

The kidney had normal architecture but contained crystals of varied morphology in the tubular lumina. Most crystals were rosettes of oxalate-like, birefringent material as seen in the respiratory tissues, but there were also a few oval to round birefringent crystals, a few lightly basophilic nonrefractive calcospheres, and rare tubules with fibrillar basophilic nonrefractive crystals (urates). The latter was associated with a granulomatous and heterophilic response.

The results of histochemical testing of the rosette crystals using von Kossa stain and Pizzolato's method were consistent with previously reported staining characteristics of calcium oxalate crystals [1, 2].

Fungal culture of the air sac identified fungal colonies consistent with* Aspergillus *section* Nigri*. Fungal identification was performed microscopically using morphologic characteristics.

## 3. Discussion

*Aspergillus spp*. are opportunistic, widely distributed fungal molds that are most commonly found in soil and other organic matter.* Aspergillus spp*. affects humans, mammals, and birds, both wild and domestic, causing aspergillosis. Aspergillosis is primarily considered an opportunistic infection, causing disease in immune compromised hosts, but can be a primary pathogen, particularly in poultry after inhalation of spores in their environment and/or feed. There is some suggestion that the predisposing features for aspergillosis in birds are instead species predilection (i.e., raptors, turkeys, penguins, waterfowl) and various stressors (i.e., environmental, extensive physical exertion) [3]. Avian aspergillosis primarily affects the respiratory system [4].

*Aspergillus fumigatus* is the most commonly reported species causing aspergillosis in human and veterinary medicine. Less commonly,* Aspergillus niger* (*A. niger*) has been associated with human diseases, such as cutaneous infections [5] and pulmonary disease [6, 7]. Definitive diagnosis of* A. niger* requires molecular assays which are unavailable for this case and, so, we will refer to the fungus cultured in this case as* Aspergillus* section* Nigri* given our level of suspicion based on morphologic features. We suspect that the* Aspergillus* section* Nigri* infection in this case has occurred as a result of living in captivity (i.e., decreased ventilation, exposure to higher concentration of fungal spores) with the prior ocular surgery perhaps being a contributing stress factor.

Calcium oxalate crystals have previously been reported in the diagnosis of an* Aspergillus spp*. infection, including* A. niger *[7]. Oxalic acid precipitates and forms insoluble calcium oxalate crystals when generated via the tricarboxylic acid cycle [8] although there is some evidence that cytoplasmic generation of oxalate genesis is also possible in this species [9]. With particular regard to* Aspergillus niger*, there is a pH-responsive transcription factor that can alter metabolism in favor of oxalate production [10].

The association of calcium oxalate crystals with* A. niger* infection is well recognized in human medicine, and it has been suggested that, even in the absence of visualized conidia, the presence of these crystals may indicate* A. niger* infection [11]. This lack of physical association between fungal elements and oxalate deposits has been reported in rare cases of human medicine, primarily within renal tubules [12]. Renal oxalosis has been postulated for ovine* Aspergillus* exposure [13] and may provide an alternative explanation for a case of renal oxalosis in a cervid [14]. However, the prevalence of oxalate crystals in veterinary cases of aspergillosis may be higher than reported. In a retrospective study, the presence of calcium oxalate crystals was assessed for 38 cases of previously diagnosed aspergillosis in mixed veterinary species, including avian [15]. Only 3 of the 38 cases were originally described as containing crystals, yet the authors found additional 11 cases that contained calcium oxalate crystals upon histologic reexamination. In this study,* A. niger, A. fumigatus, A. versicolor,* and unspeciated* Aspergillus *sp. were all associated with calcium oxalate crystal deposition. Furthermore, this study reported that oxalate crystals were mainly found in the respiratory tract (tissues with an air interface); however, rare to few crystals consistent with calcium oxalate were identified in renal tubules of approximately 25% of these cases. This renal targeting is not unanticipated, since studies in humans indicate that glomerular filtration of oxalates, combined with proximal tubular secretion, is the primary means of excretion of this substance [16].

The mechanism by which the calcium oxalate crystals become deposited in nonrespiratory tissues is unclear. Presumably oxalates are being absorbed into the bloodstream following deposition in the interstitial space by fungi and there is precipitation at other sites depending on factors such as pH, oxygen concentration, and local electrolyte levels. It is plausible that concurrent metabolic derangements were present which predisposed this particular bird to the development of widespread oxalosis. For instance, given the severity of the respiratory disease found at necropsy, there may have been a systemic change in pH and/or oxygen concentration from impaired respiratory function that permitted formation of calcium oxalate crystals at sites away from fungal hyphae. Alternatively, dehydration resulting in prerenal azotemia and accumulation of uric acid may have altered systemic pH; this is supported by the presence of urate crystals in the kidney (i.e., nephric gout) which is often a result of dehydration in avian species.

Though there are previous reports of* A. niger* in avian species—including a great horned owl [17], ostrich [18], poultry [19], falcons [20], and parrots [15]—this is the first report to identify* Aspergillus *section* Nigri *in a Eurasian eagle owl. An unusual density of thoracic lesions on radiographs, as seen here, should increase the index of suspicion for respiratory aspergillosis. The fuzzy texture and characteristic black pigment of the fungus seen on gross examination were consistent with* Aspergillus *section* Nigri* as was the abundant oxalate crystal deposition noted histologically. To our knowledge, this is the first report that finds widespread distribution of fungal-origin calcium oxalate crystals in nonrespiratory tissues in an avian or mammalian species, providing a variation in the spectrum of* Aspergillus*-oxalosis in veterinary medicine.

## Data Availability

The histological and histochemical stains used to support the findings in this study are included in the article while previously reported data used to support this case study are cited at relevant places within the text as [1–3, 5, 7, 9–20]. The specifics regarding the fungal culture used to support the findings of this study are available from the corresponding author upon request.
